# Racial disparities affect the association between gestational urinary phthalate mixtures and infant genital measures

**DOI:** 10.3389/frph.2023.1304725

**Published:** 2023-12-11

**Authors:** Meghana Varde, Roger B. Newman, Abby G. Wenzel, John R. Kucklick, Rebecca J. Wineland, John W. Brock, Michael S. Bloom

**Affiliations:** ^1^ Department of Global and Community Health, George Mason University, Fairfax, VA, United States; ^2^ Department of Obstetrics and Gynecology, Medical University of South Carolina, Charleston, SC, United States; ^3^ Hollings Marine Laboratory, National Institute of Standards and Technology, Charleston, SC, United States; ^4^ Department of Chemistry, University of North Carolina Asheville, Asheville, NC, United States

**Keywords:** anogenital distance, BKMR, penile measures, phthalates mixture, race disparity

## Abstract

**Background:**

Phthalates are ubiquitous anti-androgenic endocrine disrupting chemicals found in personal care products, medications, and many plastics. Studies have shown a racial disparity in phthalates exposure among U.S. women, which may also impact fetal development.

**Methods:**

We conducted a prospective cohort study of gestational exposure to a phthalates mixture in a racially-diverse population to determine their association with genital development. Mid-gestation (18–22 weeks) urine was collected from 152 women who self-identified as non-Hispanic Black and 158 women who self-identified as non-Hispanic White in Charleston, South Carolina between 2011 and 2014. We measured eight phthalate monoester metabolites in urine using liquid chromatography tandem-mass spectrometry. Mid-gestational penile dimensions were measured using ultrasound and anogenital distances were measured postnatally. We used Bayesian kernel machine regression to estimate the associations among the mixture of phthalate metabolites and mid-gestation penile dimensions and postnatal anogenital distance measures among singleton male (*n* = 179) and female (*n* = 131) infants, adjusted for urinary specific gravity, maternal age, body mass index, education level, cigarette smoking, and gestational age at enrollment or birth weight *z*-score.

**Results:**

We found a stronger association between greater phthalates and decreased anopenile distance among infants born to women who self-identified as Black. Mono (2-ethylhexyl) phthalate (MEHP) was the driving mixture component among Black women, and monobutyl phthalate (MBP) and monoethyl phthalate (MEP) were drivers among White women. We also identified a non-linear association between phthalates and lesser ultrasound penile volume among women who self-identified as Black with monoisobutyl phthalate (MiBP) and MBP being most important. We also found an association between greater phthalates and shorter anoclitoral distance among infants born to women who self-identified as Black, with MEP and monobenzyl phthalate (MBzP) contributing most to this association.

**Conclusion:**

Our results suggest a disparity in the association between gestational exposure to a mixture of phthalates and fetal genital development among women who self-identified as Black compared to White.

## 1. Introduction

Phthalates are ubiquitous endocrine disrupting chemicals used in personal care products and cosmetics, plastic food and beverage packaging, toys, and other consumer products (1). Human exposure to phthalates is widespread (2) and occurs via dermal absorption, ingestion, or inhalation of volatized phthalates (3). Low molecular weight phthalates are used as solvents and fragrance carriers in personal care products, such as lotions, soaps, and perfumes, including diethyl phthalate (DEP) and dibutyl phthalate (DBP). High molecular weight phthalates, like di-(2-ethylhexyl) phthalate (DEHP), tend to be used in plastics, especially polyvinyl chloride packaging (4). In the U.S., minoritized groups experience greater levels of exposure to many phthalates compared to White populations (5). Some phthalates have anti-androgenic properties in experimental studies (6) and cross the placental barrier to potentially affect a developing fetus. Gestational phthalates exposure caused male reproductive organ malformations, diminished testosterone and inhibited Leydig cell steroidogenesis in experimental animal models (7, 8), although the evidence from human testicular explant studies was mixed (9, 10). Some phthalates are shown to have estrogenic effects in experimental studies (11, 12). Phthalates can act as estrogen receptor agonists (13) and androgen receptor antagonists (14), which may result in reduced testosterone and sperm production in males (15). While most work has focused on the effects of individual phthalates, more recent studies suggest that the biological effects of phthalates may differ in the context of a mixture (16), prompting the U.S. National Academy of Sciences to call for cumulative risk assessment approaches to the endocrine disrupting effects of phthalates (17). For example, phthalate mixtures elicited meaningful dose-additive anti-androgenic effects in male rats (18–20). Another study found that gestational exposure to an environmentally-relevant mixture of phthalates [DEHP, DEP, DBP, benzyl butyl phthalate (BBP), di-isobutyl phthalate (DiBP), and diisononyl phthalate (DiNP)], was associated with decreased anogenital distance (AGD) in female mice, although at a greater dose than typically experienced by human populations (21). Furthermore, dose-response associations may be non-linear. For example, there was a stronger positive association between AGD and DEHP at low doses (0.5 µg/kg/day) than at higher doses (500,000 µg/kg/day) in gestationally-exposed male mice (22).

Results from human studies of gestational phthalates exposure and fetal genital development have been inconsistent. Previous studies have examined associations between individual phthalates and measures of AGD, the length from the anus to the genitalia, though results were discordant (3, 23). A longer AGD is a biomarker of greater fetal exposure to androgens during early pregnancy and has been correlated to reproductive health endpoints in adults (24, 25). In our previous work, we reported inverse associations between anopenile distance (APD) and maternal urinary mono(2-ethylhexyl) phthalate (MEHP), monobutyl phthalate (MBP), monobenzyl phthalate (MBzP), mono(2-ethyl-5-hydroxyhexyl) phthalate (MEHHP), mono(2-ethyl-5-oxohexyl) phthalate (MEOHP), monoethyl phthalate (MEP), monoisobutyl phthalate (MiBP), and monomethyl phthalate (MMP) among male infants (26). Among female infants, we reported a positive association between maternal urinary MBzP and anoclitoral distance (ACD), but inverse associations for maternal urinary MBP, MiBP, MEHP, MEOHP, MEHHP, MEP, and MMP with ACD (26). We also found inverse associations between maternal urinary MiBP, MBzP, MEHP, MEOHP, MEHHP, MEP, and MMP with ultrasound-derived penile volume (PV); however, a positive association between maternal urinary MBP and ultrasound PV (27). Our results suggested a differential association between gestational urinary phthalates among women who self-identified as Black (including African American) and White (26, 27), and for female and male infants. However, to our knowledge, no studies have investigated the potential effects of gestational exposure to a phthalates mixture on fetal genital development, which may differ from the effects of individual phthalates in isolation (28).

Therefore, our aim was to estimate associations between gestational exposure to a mixture of eight urinary phthalate metabolites with ultrasound-derived penile volumes and postnatal AGD measurements among mother-infant pairs. These results will help to inform risk assessments of the fetal developmental health risks of gestational phthalates exposure as called for by the National Academy of Sciences (17).

## 2. Methods

### 2.1. Study population

From 2011 to 2014, we enrolled 407 women with a viable singleton pregnancy into a birth cohort study during a routine fetal anatomic ultrasound visit between 18 and 22 weeks' gestation. Women were eligible if they were 18 years of age or older, had no obvious fetal anomalies by fetal ultrasound, agreed to identification of the fetal sex, and planned to deliver at the Medical University of South Carolina (MUSC) (26, 27, 29). Women were excluded with multiple gestations, did not want to learn the fetal sex, had fetal congenital anomalies or endocrine diagnoses, and used steroids or other medications. At enrollment, women provided a spot urine specimen and completed an interviewer-administered study questionnaire to collect information about sociodemographic and lifestyle factors. Clinical data were extracted from the electronic medical record. This study includes 310 women with live deliveries who self-identified as non-Hispanic Black (*n* = 152), including African American, or non-Hispanic White (*n* = 158) (subsequently referred to as Black and White, respectively), and had a urine phthalates analysis. We use racial grouping as a proxy for individual and societal experiences driven by ongoing historical processes based on one's identify, presumably reflecting skin pigmentation (30). All participants in this study completed written informed consent and the study protocol was approved by the MUSC Institutional Review Board.

### 2.2. Measures of postnatal anogenital distance and prenatal ultrasound penile dimensions

Measures of AGDs, the length from the anus to the genitalia, were completed within 48 h of delivery using a caliper as previously described in detail (26). Briefly, each AGD was measured in triplicate and averaged, with infants lying on their back and legs in the frog position. For males, we measured APD and anoscrotal distance (ASD) as the distance from the anterior margin of the anus to the base of the penis or to the base of the scrotum where the skin changes from smooth to rugated, respectively. For females, ACD and anofourchette distance (AFD) were measured as the distance from the anterior margin of the anus to the clitoral hood or posterior convergence of the fourchette, respectively.

Ultrasound penile length (PL) and penile width (PW) measures were made in women with male fetuses between 18 and 22 weeks gestation, as previously described in detail (27). Briefly, American Institute of Ultrasound in Medicine (AIUM)-certified sonographers used freeze-frame images and electronic calipers to measure PL from the scrotal junction to the tip of the glans, and penile width (PW) was measured mid-shaft. Each dimension was measured in triplicate and the values averaged together. Penile volume (PV) was estimated as (PW/2)^2^ *PL.

### 2.3. Urinary phthalates analysis

Urine samples were processed and frozen at −20°C immediately after collection. Specific gravity was determined at room temperature using a handheld refractometer (Atago U.S.A., Inc., Bellevue, WA, USA) (26, 27). Urinary specimens were transferred, on dry ice, to Hollings Marine Laboratory, National Institute of Standards and Technology (Charleston, SC, USA). For analysis of phthalates, urinary concentrations of eight phthalate monoester metabolites were determined using a method based on liquid chromatography coupled to tandem mass spectrometry after solid phase extraction, as previously described in detail (26), including: MBP, MiBP, MBzP, MEHP, MEOHP, MEHHP, MEP, and MMP.

Limits of detection (LOD) ranged from 0.10 ng/ml for MMP to 1.00 ng/ml for MBzP. Instrument-reported phthalate values were used for values less than the LOD without imputation to minimize bias in the regression models (31). For descriptive analysis, we corrected phthalate concentrations for urinary dilution using specific gravity as *P_c_*_ _= *P_i_*[(1.016–1)/SG_i_-1], where *P_c_*_ _= specific gravity-corrected phthalate concentration (ng/ml), *P_i_*_ _= individual urinary phthalate concentration (ng/ml); 1.016 = mean urinary specific gravity for all women in the study population, and SG_i _= individual specific gravity. However, we used urinary phthalate metabolites uncorrected for specific gravity during regression analysis, and included urine specific gravity as a covariate in the regression models (32), to prevent propagation of measurement error in phthalate values and bias that may be introduced by conventional standardization approaches in regression models.

### 2.4. Statistical analysis

We summarized the distribution of covariates, overall and according to Black and White racial identity group, and used Student's *t*-tests and Chi-square tests of the differences between the racial groups. Phthalate concentrations were natural log transformed after adding a constant (=1), to normalize the distributions and stabilize the variances prior to analysis.

We used Bayesian kernel machine regression (BKMR) (33) to estimate associations between gestational exposure to the mixture of eight urinary phthalate metabolites as a predictor and postnatal AGDs and ultrasound penile dimensions as outcomes in individual models. The BKMR approach was selected because it allows for inter-phthalate interactions and non-linear dose-response associations. Based on previous studies, we adjusted the models for maternal age (years) (34), body mass index (BMI, kg/m^2^) (35), education (“did not complete college” or “completed college or higher”) (35), cigarette smoking (never smoked or current smoker/quit smoking during pregnancy) (36), urinary specific gravity, and either gestational age at enrollment (weeks) for penile dimensions or birth weight standardized to World Health Organization (WHO) growth charts (*Z*-score) for AGDs (37). We used multiple imputation by chained equations (MICE) to create 10 datasets with missing covariates imputed for *n* = 22 and *n* = 1 for MEP and MMP (38). For each outcome, we estimated 10 individual BKMR models using the MICE imputed datasets (39, 40). We averaged the posterior inclusion probabilities (PIPs) from the 10 imputed datasets, and we visually inspected the trace plots to ensure convergence of the imputed datasets and the BKMR models. We implemented 50,000 to 200,000 iterations, removed 50% burn-in iterations, and retained 10% of the remaining chains to ensure stable estimates of the associations (39, 40).

Statistical significance was defined as *p* < 0.05 for a two-tailed hypothesis test. All analyses were conducted using R software version 4.2.2 (R Foundation for Statistical Computing, Vienna, Austria).

## 3. Results

### 3.1. Demographic, lifestyle, and clinical characteristics of the study population

Table 1 shows the distributions of demographic characteristics and infant genital measures for the *n* = 310 women included in this analysis (*n* = 152 women who identified as Black and *n* = 158 women who identified as White). The mean overall age was 27.61 years and the pre-pregnancy BMI was 29.14 kg/m^2^. Women who identified as Black were younger and had a greater BMI than women who identified as White. More women who identified as Black (77.30%) had less than a college education compared to women who identified as White (36.42%). A greater number of male infants were included in this study than female infants because only mothers carrying male fetuses were initially eligible to participate in our study. Ultrasound PL, PW, and PV were significantly greater among infants born to women who identified as Black, although with significantly lesser birth weight and shorter gestational age at delivery.

**Table 1:** Demographic characteristics and anogenital distance measures of women and offspring from Charleston, South Carolina, overall and by racial identity.

Characteristic	Overall (*n* = 310)	Black (*n* = 152)	White (*n* = 158)	*p*-value
Age (years), mean (SD)^a^	27.61 (5.64)	26.07 (5.62)	29.09 (5.26)	<0.001
Pre-pregnancy BMI (kg/m^2^), mean (SD)^a,b^	29.14 (7.14)	31.27 (7.94)	27.09 (5.56)	<0.001
Education, *n* (%)^c,d^				<0.001
< College	164 (56.16)	109 (77.30)	55 (36.42)	
≥ College	128 (43.84)	32 (22.70)	96 (63.58)	
Infant sex, *n* (%)^d^				0.452
Male	179 (57.74)	84 (55.26)	95 (60.13)	
Female	131 (42.26)	68 (44.74)	63 (39.87)	
Smoking status, *n* (%)^d,e^				0.648
Never smoked	270 (87.95)	131 (86.75)	139 (89.10)	
Current smoker^f^	37 (12.05)	20 (13.25)	17 (10.90)	
Birth measures (mm), mean (SD)				
APD^a,g^	44.47 (5.61)	44.07 (5.27)	44.80 (5.88)	0.392
ASD^a,g^	22.53 (4.81)	23.27 (4.56)	21.89 (4.95)	0.060
ACD^a,h^	33.60 (4.07)	33.59 (3.67)	33.61 (4.50)	0.979
AFD^a,h^	12.88 (2.50)	13.33 (2.50)	12.39 (2.44)	0.034
Ultrasound measures (mm), mean (SD)				
PV (mm^3^)^a,b^	48.04 (20.34)	52.79 (18.48)	43.89 (21.06)	0.003
PW^a,b^	5.23 (0.70)	5.34 (0.64)	5.13 (0.74)	0.046
PL^a,b^	6.77 (1.32)	7.19 (1.26)	6.34 (1.24)	<0.001
WHO standardized birth weight *z*-score^a^				
Male infants, mean (SD)	−0.13 (1.20)	−0.54 (1.36)	0.23 (0.92)	<0.001
Female infants, mean (SD)	−0.04 (1.17)	−0.36 (1.00)	0.31 (1.23)	0.001
Gestational age, (weeks), mean (SD)^a,i^	20.07 (0.71)	20.21 (0.65)	19.92 (0.74)	<0.001

ACD, anoclitoral distance; AFD, anofourchette distance; ASD, anoscrotal distance; APD, anopenile distance; PL, ultrasound penile length; PV; ultrasound penile volume; PW, ultrasound penile width.

^a^ Student's *t*-test.

^b^ *n* = 1 missing.

^c^ *n* = 18 missing.

^d^ Chi-Square test of independence.

^e^ *n* = 3 missing.

^f^ Current smoker or quit since learning of pregnancy.

^g^ *n* = 8 missing among male infants.

^h^ *n* = 3 missing among female infants.

^i^ Gestational age at enrollment.

Table 2 shows the distributions of specific-gravity corrected urinary phthalate metabolite concentrations. Most values exceeded the LOD for all measured phthalates (86.1–100%) The mean concentrations of MBP, MiBP, MEHP, and MEP were greater among women who identified as Black. However, the mean concentrations of MBzP, MEOHP, MEHHP, and MMP were greater among women who identified as White.

**Table 2:** Distribution of maternal urinary specific gravity-corrected phthalate concentrations (ng/ml), among women from Charleston, South Carolina, overall and by racial identity.

Phthalate	LOD	*n* (%) > LOD	Mean	SD	Minimum	Median	Maximum
Overall (*n* = 310)							
MBP	0.950	305 (98.4)	28.2	80.2	1.57	16.5	1.35 × 10^3^
MiBP	0.170	310 (100.)	18.6	38.4	1.20	10.8	6.10 × 10^2^
MBzP	0.100	304 (98.1)	44.8	3.90 × 10^2^	0.0141	12.1	6.87 × 10^3^
MEHP	0.350	297 (95.8)	6.08	14.7	0.306	2.96	211
MEOHP	0.100	310 (100.)	9.96	30.2	0.853	5.78	517
MEHHP	0.100	310 (100.)	12.8	35.3	0.960	7.08	591
MEP^a^	1.00	306 (98.7)	204	555	2.88	46.7	4.81 × 10^3^
MMP^a^	0.340	267 (86.1)	7.26	48.3	−2.75	2.10	772
Black (*n* = 152)							
MBP	0.950	152 (100.)	32.2	33.9	2.30	22.6	191
MiBP	0.170	152 (100.)	23.1	22.8	2.11	15.8	169
MBzP	0.100	152 (100.)	31.3	44.5	0.644	17.1	308
MEHP	0.350	151 (99.3)	6.37	9.68	0.454	3.34	91.7
MEOHP	0.100	152 (100.)	8.71	8.89	1.22	5.78	51.9
MEHHP	0.100	152 (100.)	11.9	13.7	0.960	7.09	84.1
MEP^a^	1.00	151 (99.3)	268	631	10.7	58.7	4.81 × 10^3^
MMP^a^	0.340	143 (94.1)	4.78	6.04	−0.049	3.01	43.2
White (*n* = 158)							
MBP	0.950	153 (96.8)	24.4	107	1.57	12.8	1.35 × 10^3^
MiBP	0.170	158 (100.)	14.2	48.6	1.20	8.28	6.10 × 10^2^
MBzP	0.100	152 (96.2)	57.9	545	0.0141	7.61	6.87 × 10^3^
MEHP	0.350	146 (92.4)	5.81	18.4	0.306	2.78	211
MEOHP	0.100	158 (100.)	11.2	41.5	0.853	5.78	517
MEHHP	0.100	158 (100.)	13.7	47.6	1.29	7.06	591
MEP	1.00	155 (98.1)	142	466	2.88	29.5	3.90 × 10^3^
MMP	0.340	124 (78.5)	9.63	67.3	−2.75	1.67	772

LOD, limit of detection; MBP, monobutyl phthalate; MBzP, monobenzyl phthalate; MEHHP, mono(2-ethyl-5-hydroxyhexyl) phthalate; MEHP, mono(2-ethylhexyl) phthalate; MEOHP, mono(2-ethyl-5-oxohexyl) phthalate; MEP, monoethyl phthalate; MiBP, monoisobutyl phthalate; MMP, monomethyl phthalate; SD, standard deviation.

^a^ *n* = 1 missing.

### 3.2. Associations among a mixture of urinary phthalate metabolites, AGDs, and penile dimensions among male infants

**Figure 1:**
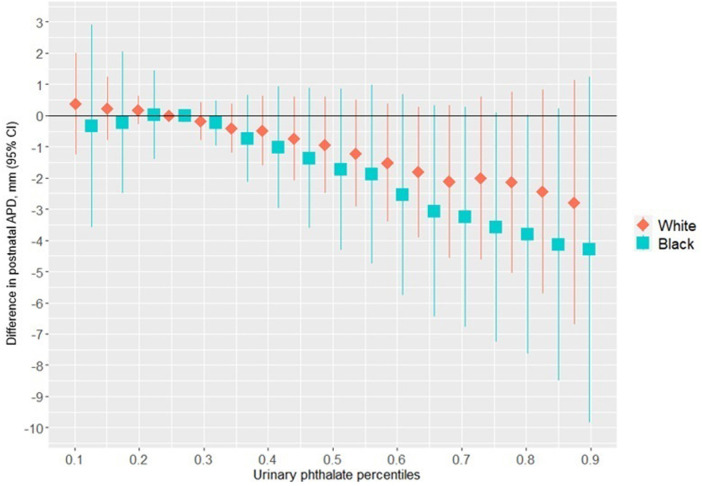
Differences in postnatal APD associated with percentiles of a urinary phthalate metabolites mixture (with the 25th percentile as the reference), adjusted for maternal specific gravity, maternal age, maternal BMI, maternal cigarette smoke, maternal education level, and wHO standardized birth weight as *z*-scores, among male infants born to women in Charleston, South Carolina, by racial identity (*n* = 171).

**Figure 2:**
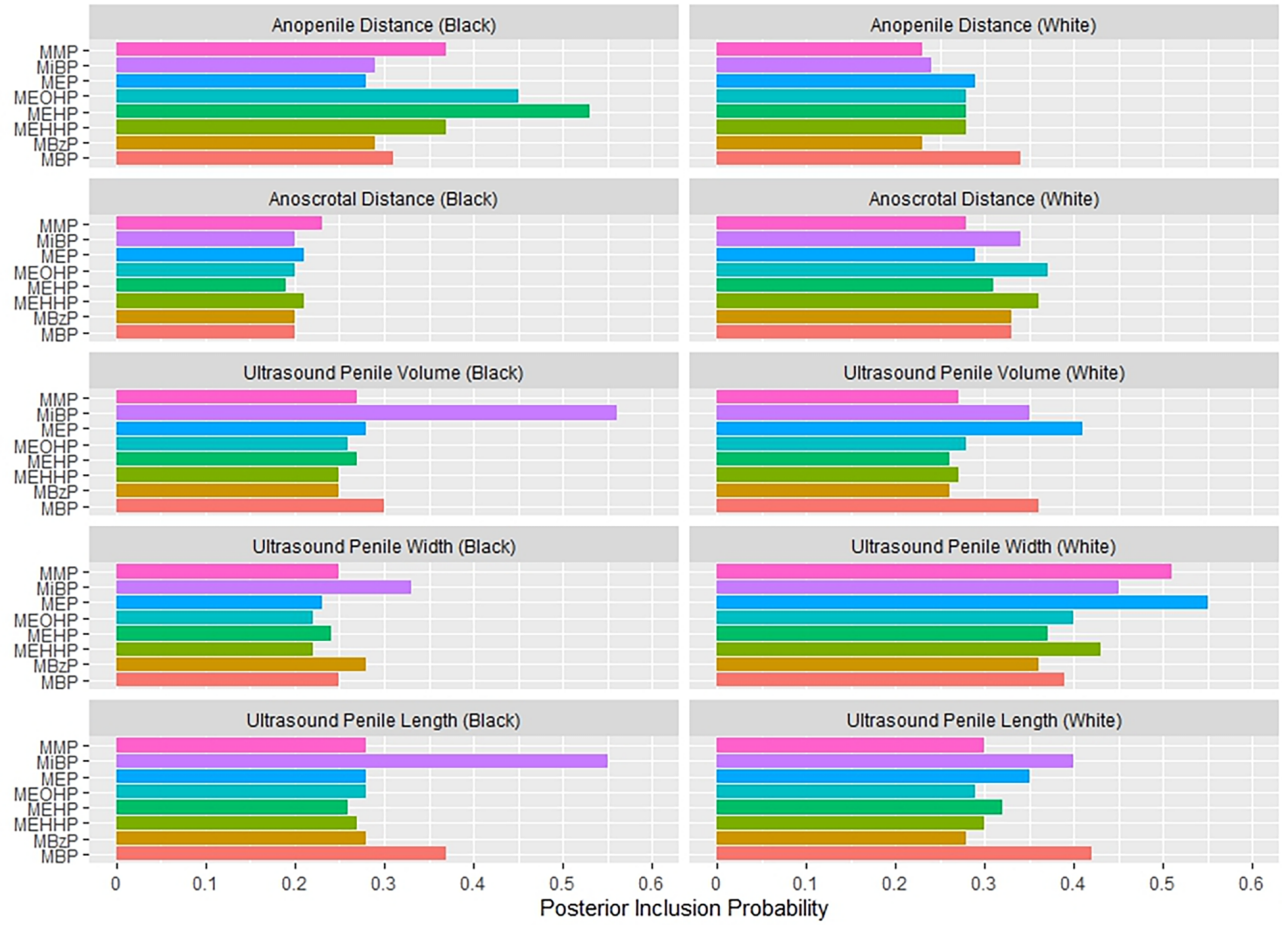
Posterior inclusion probabilities for urinary phthalate metabolite predictors of prenatal ultrasound penile dimensions and postnatal anogenital distance measures among male infants born to women in Charleston, South Carolina, by racial identity (*n* = 179).

Figure 1 shows the association between the overall phthalates mixture and APD, stratified by maternal racial identity. Greater levels of the urinary phthalate mixture were associated with lesser APD in the Black and White groupings although the association was stronger among women who identified as Black. For example, relative to the 25th percentile of the urinary phthalates concentration distribution, APD was −4.29 mm [95% credible interval (CI): −9.83 mm, 1.24 mm] shorter at the 90th percentile among the Black male newborns, but −2.78 mm (95% CI: −6.70 mm, 1.13 mm) shorter at the 90th percentile in the White male newborns. As shown in Figure 2 and Supplementary Table S1, the association between the gestational urinary phthalate metabolite mixture and APD was driven primarily by MEHP and MEOHP among women who identified as Black, whereas MBP was most important among women who identified as White.

The univariate exposure response plots in Supplementary Figure S1 show the associations between individual urinary phthalate metabolite concentrations with APD in the Black male newborns, fixing all other urinary phthalate metabolite concentrations at the 50th percentile. There was a nonlinear negative association suggested between both MEHP and MEOHP and APD in the women who identified as Black. Supplementary Figure S2 shows the associations between an interquartile range difference in the urinary concentrations of each phthalate metabolite and APD, with the other urinary phthalate metabolite concentrations fixed at the 25th percentile, 50th percentile, and 75th percentile, among women who identified as Black; there was no evidence of heterogeneity of the associations.

Supplementary Figure S3 shows the univariate exposure response plot between the individual urinary phthalate metabolite concentrations and APD among the white male newborns, fixing all other urinary phthalate metabolite concentrations at the 50th percentile. There was a negative linear association between MBP and APD, a modest nonlinear negative association suggested between greater MEHP and APD, and a positive linear association between MiBP and APD. Supplementary Figure S4 shows the associations between an interquartile range difference in the urinary concentrations of each phthalate metabolite and APD, with the other phthalate metabolite concentrations fixed at the 25th percentile, 50th percentile, and 75th percentile among women who identified as White; there was no evidence of heterogeneity of the associations.

Differences in ASD were small and imprecise at the 90th percentile compared to the 25th percentile of the urinary phthalates mixture distribution; approximately 1.70 mm (95% CI: −1.61 mm, 5.58 mm) among women who identified as Black and −1.62 mm (95% CI: −6.00 mm, 1.63 mm) among women who identified as White (Supplementary Figure S5).

**Figure 3:**
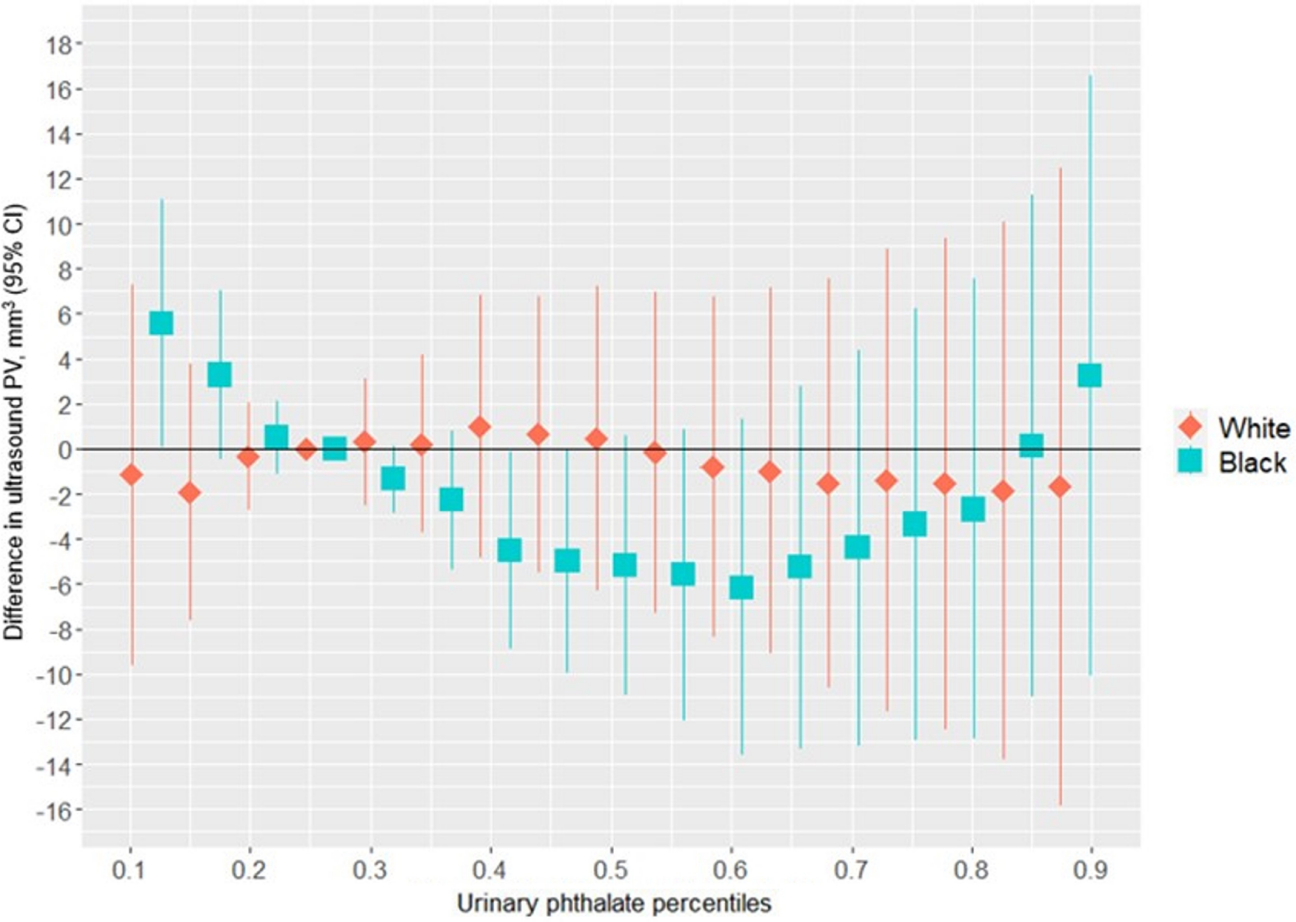
Differences in ultrasound PV associated with percentiles of a urinary phthalate metabolites mixture (with the 25th percentile as the reference), adjusted for maternal specific gravity, maternal age, maternal BMI, maternal cigarette smoke, maternal education level, and gestational age at enrollment, among male infants born to women in Charleston, South Carolina, by racial identity (*n* = 178).

Figure 3 suggests a non-linear association between the urinary phthalates mixture and ultrasound-derived PV among women reporting a Black identity. Relative to the 25th percentile, PV was greatest at the 10th percentile (5.57 mm^3^; 95%CI: 0.07 mm^3^, 11.07 mm^3^) and the 90th percentile (3.25 mm^3^; 95% CI: −10.11 mm^3^, 16.60 mm^3^), and least at the 60th percentile (−6.14 mm^3^; 95% CI: −13.61 mm^3^, 1.34 mm^3^). In contrast, no association was suggested among the women reporting a white racial identity. As shown in Figure 2 and Supplementary Table S1, the association between the gestational urinary phthalate metabolites mixture and PV was driven primarily by MiBP among women who identified as Black.

Supplementary Figure S6 shows the univariate exposure responses between urinary phthalate metabolites and ultrasound-derived PV among infants born to women who identified as Black. There was a nonlinear “U-shaped” association between MiBP and mid-gestation fetal ultrasound PV, although the associations appeared to be null for the other urinary phthalate metabolites. There was no evidence of heterogeneity in the associations between urinary phthalate metabolites and ultrasound-derived PV among women who identified as Black (Supplementary Figure S7). Similar non-linear trends were suggested for the association between the urinary phthalate metabolites mixture and ultrasound measured PW (Supplementary Figure S8) and ultrasound measured PL (Supplementary Figure S9), although the differences were small and close to the null hypothesis.

### 3.3. Associations between the mixture of urinary phthalate metabolites and AGDs among female infants

**Figure 4:**
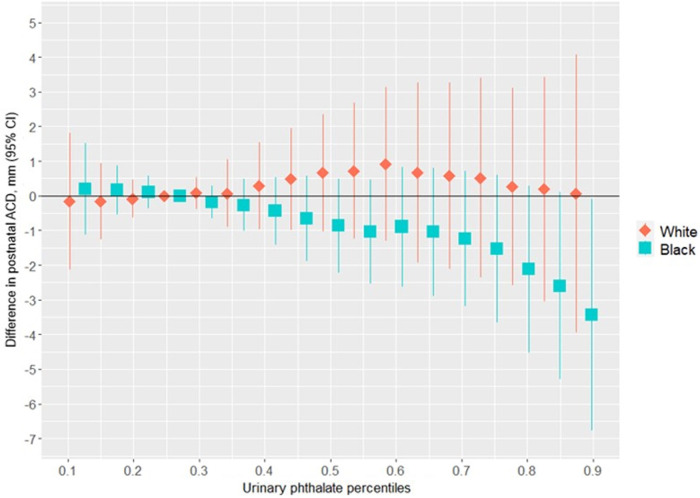
Differences in postnatal ACD associated with percentiles of a urinary phthalate metabolites mixture (with the 25th percentile as the reference), adjusted for maternal specific gravity, maternal age, maternal BMI, maternal cigarette smoke, maternal education level, and birth weight *z*-score, among female infants born to women in Charleston, South Carolina, stratified by racial identity (*n* = 128).

**Figure 5:**
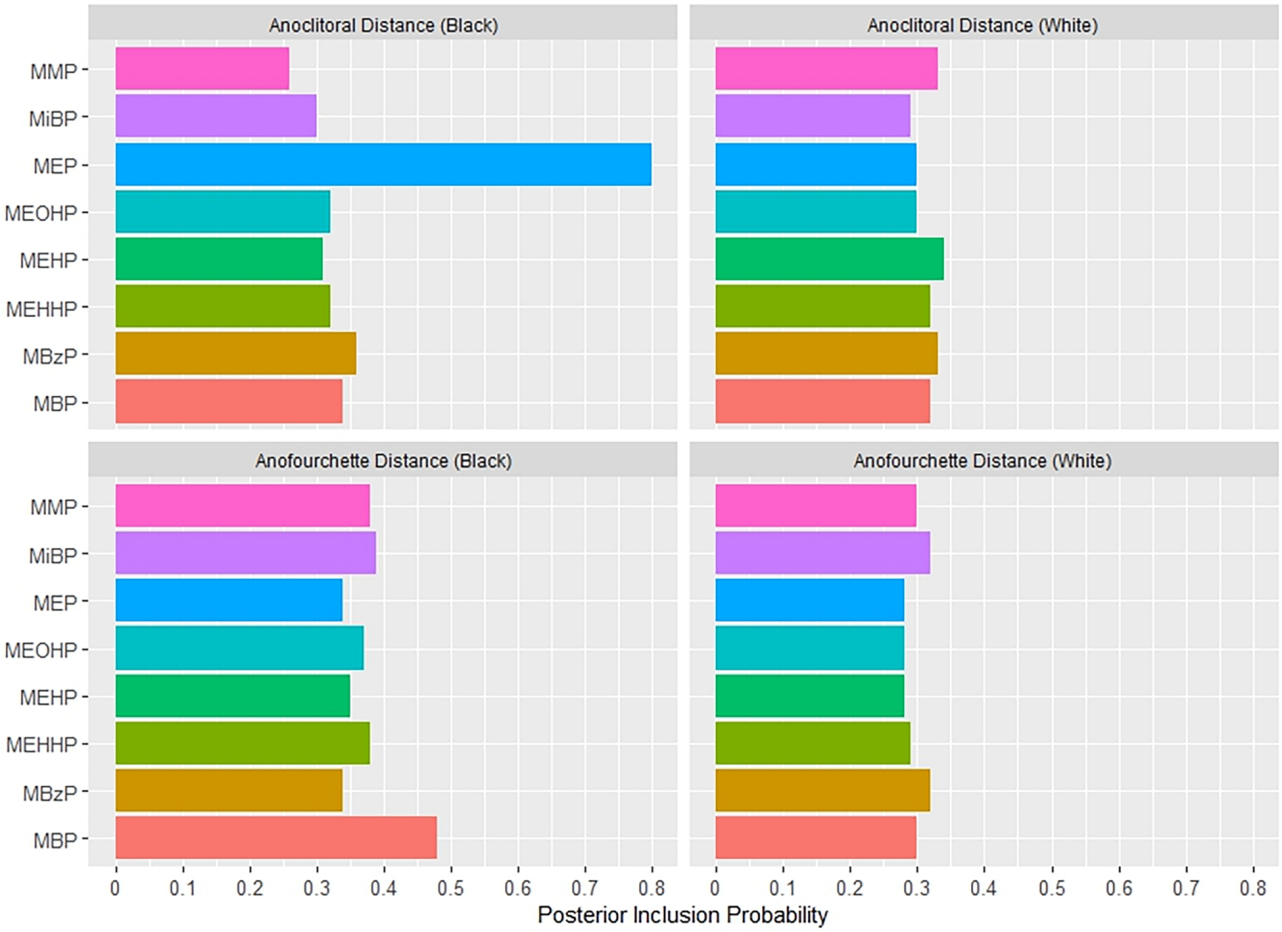
Posterior inclusion probabilities for urinary phthalate metabolite predictors of postnatal anogenital distance measures among female infants born to women in Charleston, South Carolina, by racial identity (*n* = 131).

Figure 4 shows the association between the overall phthalates mixture and ACD, stratified by racial identity. Greater levels of the urinary phthalate metabolite mixture were associated with lesser ACD in women who identified as Black, but not in women who identified as White. For example, relative to the 25th percentile of the urinary phthalate metabolites concentration distribution, ACD was −3.40 mm (95% CI: −6.78 mm, −0.08 mm) shorter at the 90th percentile in the infants of women who identified as Black, but with little difference in the infants of women who identified as White (0.06 mm; 95% CI: −3.95 mm, 4.07 mm). As shown in Figure 5 and Supplementary Table 2, the association between the gestational urinary phthalate metabolite mixture and ACD was driven primarily by MEP among women who identified as Black.

Supplementary Figure S10 shows the univariate exposure responses between urinary phthalate metabolites and postnatal ACD among female infants born to women who identified as Black. There was an inverse association for MEP with ACD and a positive association for MBzP and ACD, with other phthalates fixed at the 50th percentile concentrations. There was no evidence of heterogeneity in the associations between urinary phthalate metabolites and postnatal ACD among women who identified as Black (Supplementary Figure S1^1^).

Supplementary Figure S12 shows the association between the overall phthalates mixture and AFD, stratified by racial identity. Differences in AFD were small and imprecise at the 90th percentile compared to the 25th percentile of the urinary phthalate metabolites mixture distribution; approximately 0.77 mm (95% CI: −2.44 mm, 3.97 mm) among women who identified as Black and 0.14 mm (95% CI: −1.90 mm, 2.19 mm) among women who identified as White.

## 4. Discussion

### 4.1. Key findings

In this prospective birth cohort study, we found that greater maternal urinary concentrations of a phthalates mixture was associated with lesser APD in male infants. This change in the APD is considered a feminization or anti-androgenic effect. Among women who identified as Black, the association with APD was driven mostly by MEHP and MEOHP, but among women who identified as white the association was driven primarily by MBP. There was a non-linear association between gestational exposure to the phthalates mixture and a lesser ultrasound-derived mid-trimester PV among women who identified as Black, primarily driven by MiBP. Among women who identified as Black, we also found an inverse association of maternal urinary phthalates with ACD in female infants, which was driven primarily by MEP. The shorter ACD in female infants in association with a greater urinary phthalate mixture concentration would also suggest a feminizing effect of such exposure. These results show a racial disparity in the associations between infant genital measures and gestational exposure to a mixture of urinary phthalate metabolites in a racially-diverse population.

### 4.2. Associations between gestational phthalates exposure, AGDs, and penile dimensions in male infants

Several studies have previously investigated associations between gestational exposure to individual phthalates and male AGDs in the offspring. Many of these studies have identified inverse associations between metabolites of DEHP, but with mixed results for other phthalates (3). However, few previous studies have estimated associations between gestational exposure to a mixture of phthalates and male AGDs. A small study in Mexico (41) found an inverse association between APD and MEHP (mean difference = −0.025 mm per µg/l, *p*-value = 0.840) and with the sum of MEHP, MBzP, MEP, and MBP (mean difference = −0.191 mm per µg/l, *p*-value = 0.037). In their seminal work, Swan and colleagues (42) reported a decrease in child anogenital index (i.e., AGD divided by body weight) associated with greater gestational urinary concentration of a phthalate summary score incorporating MBP, MBzP, MEP, and MiBP (mean difference = −0.095 per log_10_ ng/ml; 95% CI: −0.17, −0.03; *p*-value = 0.009) in the multi-city U.S.-based Study of Future Families (SFF) birth cohort. We found similar inverse associations between a mixture of maternal urinary phthalate metabolites and APD in offspring using BKMR, an approach that does not place strong assumptions on the additivity of the component phthalates and allows for non-linear dose-response associations.

Previously, we reported associations between maternal urinary concentrations of individual phthalate metabolites and AGD measures in male infants from this same study population (26). We found inverse associations between APD and MBP, MiBP, MBzP, MEHP, MEOHP, MEHHP, MEP, and MMP. The strongest associations were with MEHP [mean difference = −2.07 mm per log_e_ ng/ml, 95% confidence interval (CI): −4.05, −0.08] and MEOHP (mean difference = −1.45 mm per log_e_ ng/mL, 95% CI: −3.41, 0.52) in the Black grouping, and MBP (*β* = −1.47 mm per log_e_ ng/ml, 95% CI: −3.13, 0.18) and MEHP (*β* = −1.23 mm per log_e_ ng/ml, 95% CI: −3.18, 0.73) in the White grouping (26). In our earlier work, a change in MEHP and MEOHP concentrations from the 25th percentile to the 90th percentile correspond to APD differences of −2.80 mm and −2.60 mm, respectively, among the Black grouping (i.e., vs. −4.29 mm for the mixture in our current work), and APD differences of −2.18 mm and −1.51 mm with MBP and MEHP, respectively among the White grouping (i.e., vs. −2.78 mm for the mixture in our current work). Thus, we identified similarly important urinary phthalates, but generally stronger associations with APD and a clearer racial disparity in the context of a phthalates mixture than when considered as individual phthalates. However, in the current study, there was no evidence of synergy between metabolites in the phthalates mixture with respect to APD.

Contradictory to our *a priori* hypothesis, we found small positive and negative differences in the associations between the urinary phthalates mixture and ASD in the Black and White racial groupings, respectively. We also found positive and negative associations between individual urinary phthalates and ASD in our previous work (26), although without a consistent pattern. The mechanism driving the discordant APD and ASD results is unclear, but may in part reflect unmeasured associations with scrotal volume, a hypothesis that we were unable to test in our study. A future study that incorporates scrotal volume measures and comprehensive psychosocial stress information will be necessary to confirm our results.

Few previous studies have estimated associations between gestational urinary phthalates exposure and penile dimensions among offspring (43). The aforementioned SFF reported inverse associations between a child's PW and greater maternal urinary MEHP (mean difference = −0.78 mm per log_10_ ng/ml, *p*-value = 0.005), MEHHP (mean difference = −0.53 mm per log_10_ ng/ml, *p*-value = 0.080), and the sum of DEHP metabolites (mean difference = −0.57 mm per log_10_ ng/ml, *p*-value = 0.072) among 106 mother-infant pairs (43). We previously reported both positive and inverse associations between individual maternal urinary phthalate metabolites and fetal ultrasound PL, PW, and PV that differed according to maternal racial identity, in the same study population used here (27). The associations for maternal urinary MiBP and the sum of DBP metabolites (MBP and MiBP) showed negative and positive associations with fetal ultrasound PL in our prior work, among the women who identified as Black and White, respectively. In contrast, maternal urinary MEHP, MEHHP, MMP, and the sum of DEHP metabolites (MEHP, MEOHP, and MEHHP) had mixed and discordant associations with fetal ultrasound measured PW in our prior work, among women who identified as Black and White. In our prior study, the sum of DBP metabolites was also associated with lesser fetal ultrasound-derived PV among women who identified as Black (mean difference = −1.71 mm^3^ per log_e_ ng/ml, 95% CI −6.39, 2.96) and greater fetal ultrasound-derived PV among women in the White racial grouping (mean difference = 3.84 mm^3^ per log_e_ ng/ml, 95% CI: −0.62, 8.29) (27). In contrast to our prior work, our current results in the same study population suggested a non-linear “U-shaped” dose-response association between gestational exposure to the mixture of eight urinary phthalate metabolites and fetal ultrasound-derived PV among the women who identified as Black, without an association among the women who identified as White. We did not find evidence of synergy between component phthalates within the mixture. These new results underscore the importance of evaluating gestational phthalates exposure in a mixture to identify non-linear dose-response patterns and to disentangle potentially complex environmental reproductive health race disparities.

### 4.3. Associations between gestational phthalates and AGDs in female infants

Few investigators have reported on associations between gestational urinary phthalate exposure and AGDs among female infants (26, 44). The Maternal-Infant Research on Environmental Chemicals (MIREC) study, a prospective birth cohort study of 196 mother-infant pairs in Canada, reported inverse (mean difference = −1.24 mm per log_e_ µg/l; 95% CI: −1.91, −0.57; *p*-value = 0.0004) and positive (mean difference = 0.65 mm per log_e_ µg/l; 95% CI: 0.12, 1.18, *p*-value = 0.02) associations for ACD with first-trimester maternal urinary MBzP and MEP concentrations, respectively, although without a significant association for AFD (23). However, the study population consisted mostly (>90%) of women who identified as White. Another multi-city U.S. birth cohort study of 373 mother infant pairs, The Infant Development and Environment Study (TIDES) found no association between 12 first-trimester maternal urinary phthalates and ACD or AFD, however, the results were not stratified by racial identity although 33.6% of the study population identified as non-White (44).

In our previous work in the same study population used here, we found statistically significant differences in the associations between ACD and maternal urinary MEP and the sum of urinary DBP metabolites among the female newborns of women who identified as Black (mean difference = −1.13 mm per log_e_ ng/ml; 95% CI: −1.90, −0.35 and mean difference = −0.77 mm per log_e_ ng/ml; 95% CI: −2.06, 0.51, respectively) as well as among the female newborns of women who identified as White (mean difference = 0.63; 95% CI: −0.42, 1.68 and mean difference = 1.22 mm; 95% CI: −0.66, 3.09, respectively) (26). In our earlier work, a change in MEP and the sum of DBP metabolite concentrations from the 25th percentile to the 90th percentile corresponded to ACD differences of −2.86 mm and −1.08 mm, respectively, among the Black grouping (i.e., vs. −3.40 mm for the mixture in our current work), and ACD differences of +1.33 mm for MEP and +1.80 mm and the sum of DBP, among the White grouping (i.e., vs. 0.06 mm for the mixture in our current work). We did not find evidence of synergism among mixture components. In the current study we found that MEP was an important driver of an inverse, potentially non-linear, association between the mixture of maternal urinary phthalate metabolites and ACD among women who identified as Black, but not for DBP metabolites (MBP and MiBP). The associations between the phthalates mixture and ACD appeared to be stronger than with individual phthalates among the Black grouping, whereas the estimate for the White grouping was closer to the null hypothesis. While our null results for ACD among the White racial identity grouping is consistent with the TIDES results (44), they differ from the individual phthalate exposure model results reported by MIREC (23). The associations between maternal urinary phthalates and offspring AFD have been consistently null, using individual maternal urinary phthalates as predictors in prior studies and a mixture of maternal urinary phthalate metabolites in the current study. The reason for discordant effect estimates between urinary phthalates and measures of ACD and AFD is unclear and results of experimental studies of gestational exposure to endocrine disrupting chemicals and female AGDs is mixed (24). A future study that measures fourchette-clitoral distance may offer further insight into the nature of the discrepancy.

### 4.4. Potential mechanisms

We found gestational phthalate mixtures tended to have stronger associations with infant genital measures among mothers who self-identified as Black compared to White. The racial disparity might be attributed in part to differences in dose levels as women in the Black racial grouping had greater urinary MBP, MiBP, MEHP, and MEP concentrations. However, women in the Black and White groupings had similar MEOHP concentrations. Unaccounted differences in maternal factors (24), including allostatic load and structural racism (45) may also play a role in “potentiating” gestational phthalate exposures. A future study that incorporates comprehensive psychosocial stress and lived experiences data will be necessary for a more definitive interpretation of the results.

### 4.5. Strengths and limitations

Our study has several notable strengths. Most saliently, we used a mixtures-based approach to estimate the associations of infant genital measures with simultaneous gestational exposure to eight urinary phthalate metabolites, an approach that more closely approximates “real world” exposure scenarios compared to that achieved using single phthalate predictor models in previous studies (28). We implemented BKMR, which allows for interactions among the phthalate mixture components and for non-linear associations with the study endpoints (33). Because our study population included a substantial proportion of women who self-identified as Black, we were also able to conduct a stratified analysis to estimate racial disparities in the associations between gestational exposure to a mixture of phthalates and fetal genital measures. We adjusted regression models for important confounding factors and all outcome measures were performed in triplicate, using a standardized protocol, to ensure quality control and minimize outcome misclassification. Finally, our prospective birth cohort design ensured temporality between mid-gestation exposure to a phthalates mixture and postnatal genital measurements among infants.

There are also important limitations to this study. The masculinization programming window (MPW) that governs fetal genital development is believed to occur primarily between 8 and 14 weeks' gestation (46), so our urine collection at 18–22 weeks' gestation may have misclassified exposure in some participants with a bias towards the null hypothesis. Phthalates have a short half-life *in vivo*, on the order of hours, and tend to vary across time within person, which may have further misclassified exposure in some women (47, 48). We also considered a limited panel of eight phthalate metabolites, which were highly prevalent in the U.S. population at the time of our study. However, additional phthalates found in common exposure sources, such as personal care products, may have biased our study results, so a future study with a more comprehensive phthalates mixture is necessary to confirm our results (49).

## 5. Conclusions

We found mostly inverse associations between gestational exposure to a mixture of maternal urinary phthalates and genital measure outcomes except for the measures of ASD and AFD, especially in male and female infants born to women who self-identified as Black. We also found a “U-shaped” association between ultrasound-derived PV and a mixture of phthalates among male infants in the Black racial grouping. However, the racial groupings had small sample sizes and wide credible intervals. Collectively, our results suggest a potential feminizing, anti-androgenic effect of phthalate mixture exposure during gestation. In addition, we also identify a potential racial disparity in the association between gestational phthalate exposure and fetal genital development. To our knowledge, this is the first study to report on the associations between gestational exposure to a mixture of urinary phthalate metabolites and fetal genital developmental measures among a racially diverse population. Further studies should estimate the associations between gestational exposure to a more comprehensive mixture of phthalates with multiple specimen collections across pregnancy to provide more definitive evidence for policy makers and regulators in order to guide appropriate interventions.

## Data Availability

The raw data supporting the conclusions of this article will be made available by the authors, without undue reservation.
