# Supplementary material for: Racial disparities affect the association between gestational urinary phthalate mixtures and infant genital measures
Source: Front Reprod Health. 2023 Dec 11;5:1304725. doi: 10.3389/frph.2023.1304725 (PMC10749323; doi:10.3389/frph.2023.1304725)

Supplementary Material

Supplementary Table S1. Posterior inclusion probabilities for urinary phthalate metabolite predictors of prenatal ultrasound penile dimensions and postnatal for anogenital distance measures among male infants born to women in Charleston, South Carolina, by racial identity

| PIPs | | | | | | | | | | |
| --- | --- | --- | --- | --- | --- | --- | --- | --- | --- | --- |
| Phthalate | APD | APD | ASD | ASD | PL | PL | PV | PV | PW | PW |
|  | Black | White | Black | White | Black | White | Black | White | Black | White |
|  | (n=79) | (n=92) | (n=79) | (n=92) | (n=83) | (n=95) | (n=83) | (n=95) | (n=83) | (n=95) |
| MBP | 0.31 | **0.34** | 0.20 | 0.33 | **0.37** | **0.42** | 0.30 | 0.36 | 0.25 | 0.39 |
| MiBP | 0.29 | 0.24 | 0.20 | 0.34 | **0.55** | **0.40** | **0.56** | 0.35 | **0.33** | 0.45 |
| MBzP | 0.29 | 0.23 | 0.20 | 0.33 | 0.28 | 0.28 | 0.25 | 0.26 | 0.28 | 0.36 |
| MEHP | **0.53** | 0.28 | 0.19 | 0.31 | 0.26 | 0.32 | 0.27 | 0.26 | 0.24 | 0.37 |
| MEOHP | **0.45** | 0.28 | 0.20 | **0.37** | 0.28 | 0.29 | 0.26 | 0.28 | 0.22 | 0.40 |
| MEHHP | 0.37 | 0.28 | 0.21 | **0.36** | 0.27 | 0.30 | 0.25 | 0.27 | 0.22 | 0.43 |
| MEP | 0.28 | 0.29 | 0.21 | 0.29 | 0.28 | 0.35 | 0.28 | **0.41** | 0.23 | **0.55** |
| MMP | 0.37 | 0.23 | 0.23 | 0.28 | 0.28 | 0.30 | 0.27 | 0.27 | 0.25 | **0.51** |

NOTE: Bold indicates important mixture components

Abbreviations: APD, anopenile distance; ASD, anoscrotal distance; MBP, monobutyl phthalate; MBzP, monobenzyl phthalate; MEHHP, mono(2-ethyl-5-hydroxyhexyl) phthalate; MEHP, mono(2-ethylhexyl) phthalate; MEOHP, mono(2-ethyl-5-oxohexyl) phthalate; MEP, monoethyl phthalate; MiBP, monoisobutyl phthalate; MMP, monomethyl phthalate; PIP, posterior inclusion probability; PL, ultrasound penile length; PV, ultrasound penile volume; PW, ultrasound penile width

Supplementary Table S2. Posterior inclusion probabilities for urinary phthalate metabolite predictors of postnatal anogenital distance measures among female infants born to women in Charleston, South Carolina, by racial identity

| PIPs | | | | |
| --- | --- | --- | --- | --- |
| Phthalate | ACD | ACD | AFD | AFD |
|  | Black | White | Black | White |
|  | (n=67) | (n=61) | (n=67) | (n=61) |
| MBP | 0.34 | 0.32 | **0.48** | 0.30 |
| MiBP | 0.30 | 0.29 | 0.39 | 0.32 |
| MBzP | 0.36 | 0.33 | 0.34 | 0.32 |
| MEHP | 0.31 | 0.34 | 0.35 | 0.28 |
| MEOHP | 0.32 | 0.30 | 0.37 | 0.28 |
| MEHHP | 0.32 | 0.32 | 0.38 | 0.29 |
| MEP | **0.80** | 0.30 | 0.34 | 0.28 |
| MMP | 0.26 | 0.33 | 0.38 | 0.30 |

NOTE: Bold indicates important mixture components

Abbreviations: ACD, anoclitoral distance; AFD, anofourchette distance; MBP, monobutyl phthalate; MBzP, monobenzyl phthalate; MEHHP, mono(2-ethyl-5-hydroxyhexyl) phthalate; MEHP, mono(2-ethylhexyl) phthalate; MEOHP, mono(2-ethyl-5-oxohexyl) phthalate; MEP, monoethyl phthalate; MiBP, monoisobutyl phthalate; MMP, monomethyl phthalate; PIP, posterior inclusion probability

Supplementary Figure S1. Univariate exposure response plots between urinary phthalate metabolites and postnatal APD, adjusted for maternal specific gravity, maternal age, maternal BMI, maternal cigarette smoke, maternal education level, and birth weight z-score among male infants born to women who identified as Black, in Charleston, South Carolina (n=79)


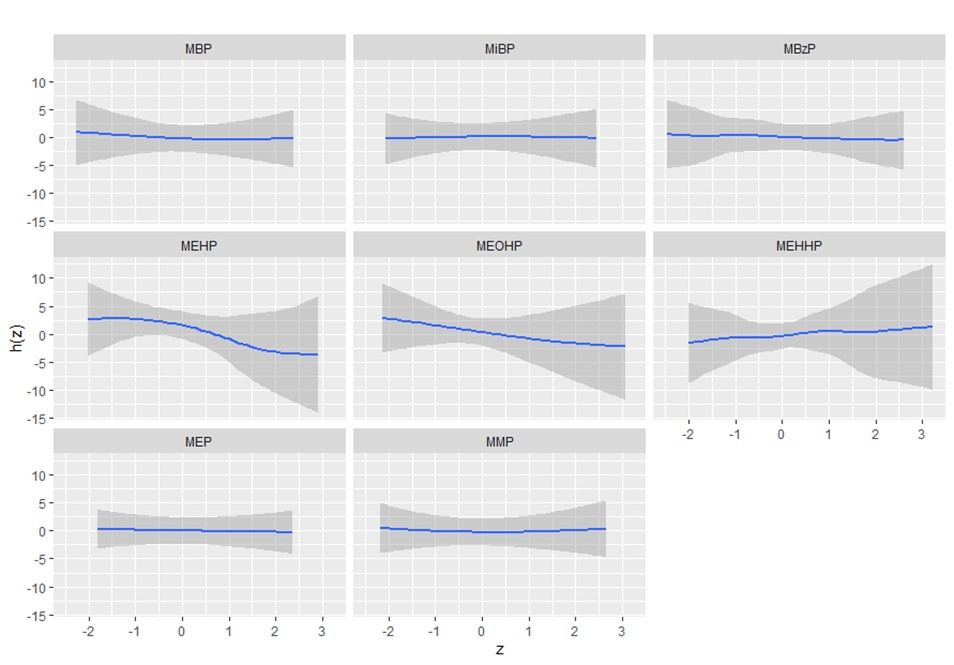


Supplementary Figure S2. Associations of a one-IQR difference between individual urinary phthalate metabolites and postnatal APD with all other phthalates held constant at the 25^th^, 50^th^, and 75^th^ percentiles, adjusted for maternal specific gravity, maternal age, maternal BMI, maternal cigarette smoke, maternal education level, and birth weight z-scores among male infants born to women who identified as Black, in Charleston, South Carolina (n=79)


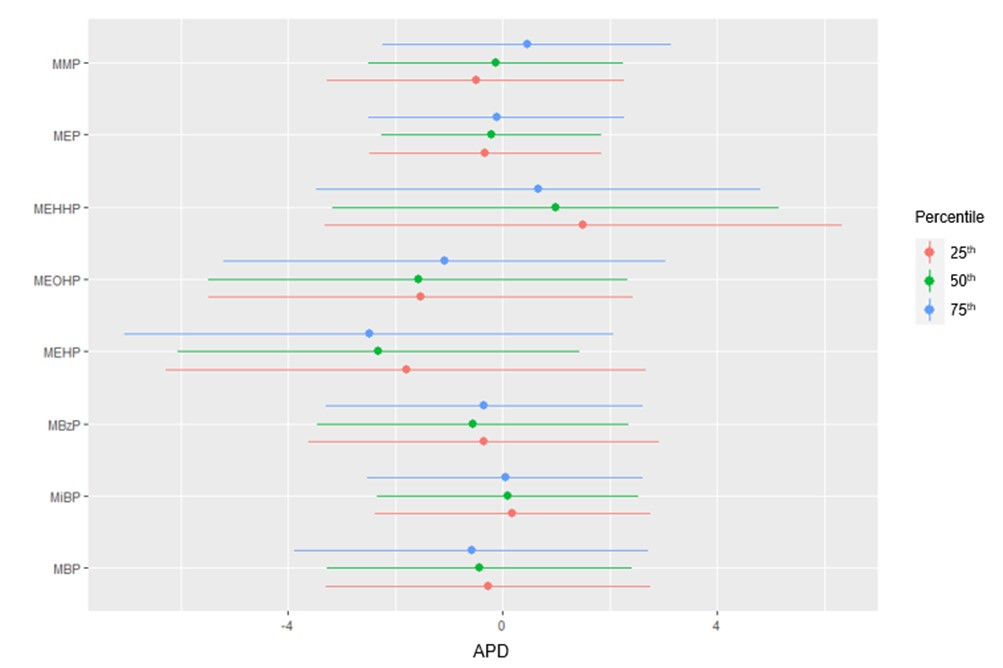


Supplementary Figure S3. Univariate exposure response plots between urinary phthalate metabolites and postnatal APD, adjusted for maternal specific gravity, maternal age, maternal BMI, maternal cigarette smoke, maternal education level, and birth weight z-score among male infants born to women who identified as White, in Charleston, South Carolina (n=92)


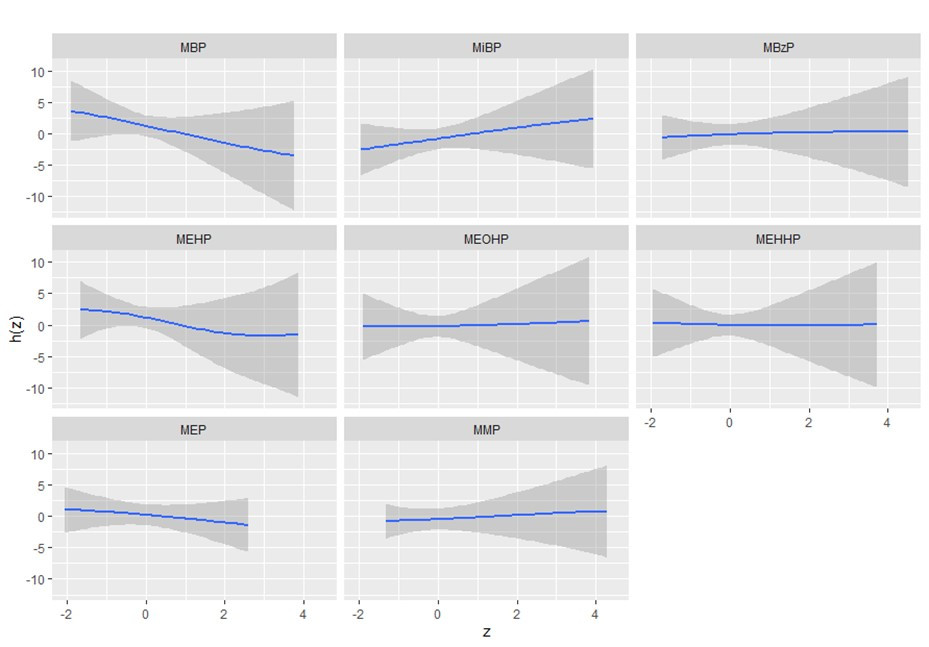


Supplementary Figure S4. Associations of a one-IQR difference between individual urinary phthalate metabolites and postnatal APD with all other phthalates held constant at the 25^th^, 50^th^, and 75^th^ percentiles adjusted for maternal specific gravity, maternal age, maternal BMI, maternal cigarette smoke, maternal education level, and birth weight z-scores among male infants born to women who identified as White in Charleston, South Carolina (n=92)


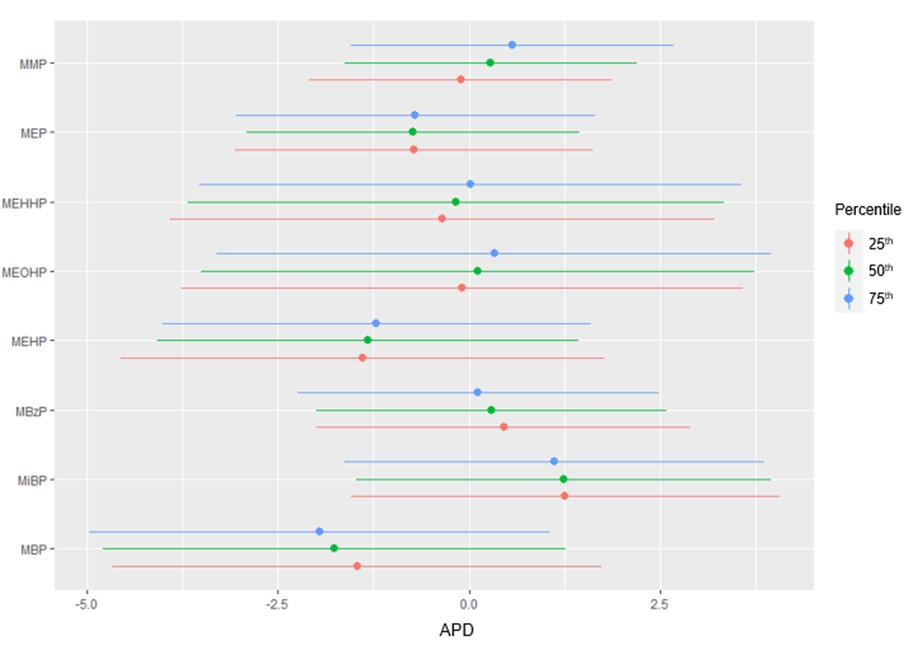


Supplementary Figure S5. Differences in postnatal ASD associated with percentiles of a urinary phthalate metabolites mixture (with the 25^th^ percentile as the reference group), adjusted for maternal specific gravity, maternal age, maternal BMI, maternal cigarette smoke, maternal education level, and birth weight z-score, among male infants born to women in Charleston, South Carolina, by racial identity (n=171)


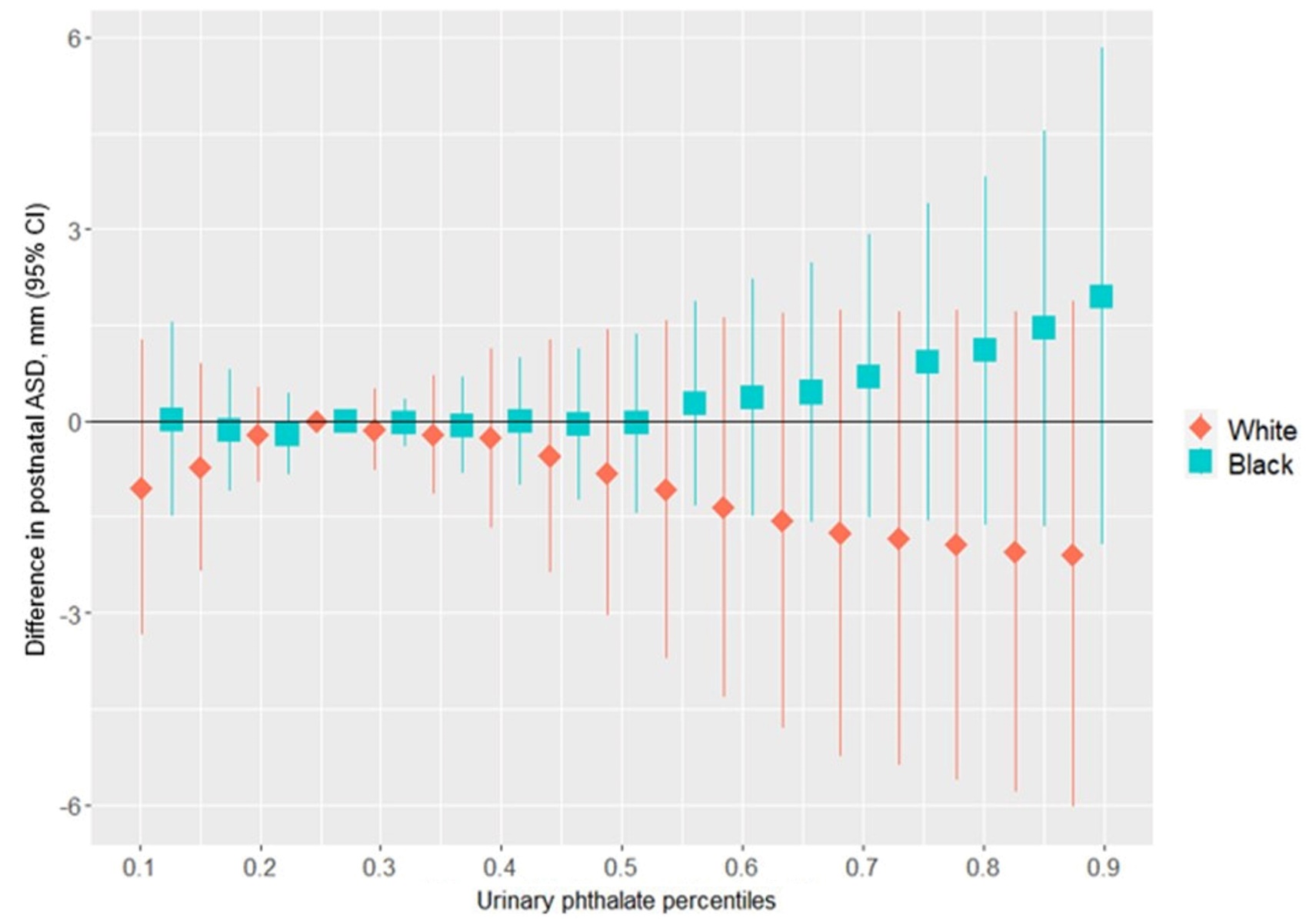


Supplementary Figure S6. Univariate exposure response plots between urinary phthalate metabolites and ultrasound PV, adjusted for maternal specific gravity, maternal age, maternal BMI, maternal cigarette smoke, maternal education level, and gestational age at enrollment, among male infants born to women who identified as Black, in Charleston, South Carolina (n=83)


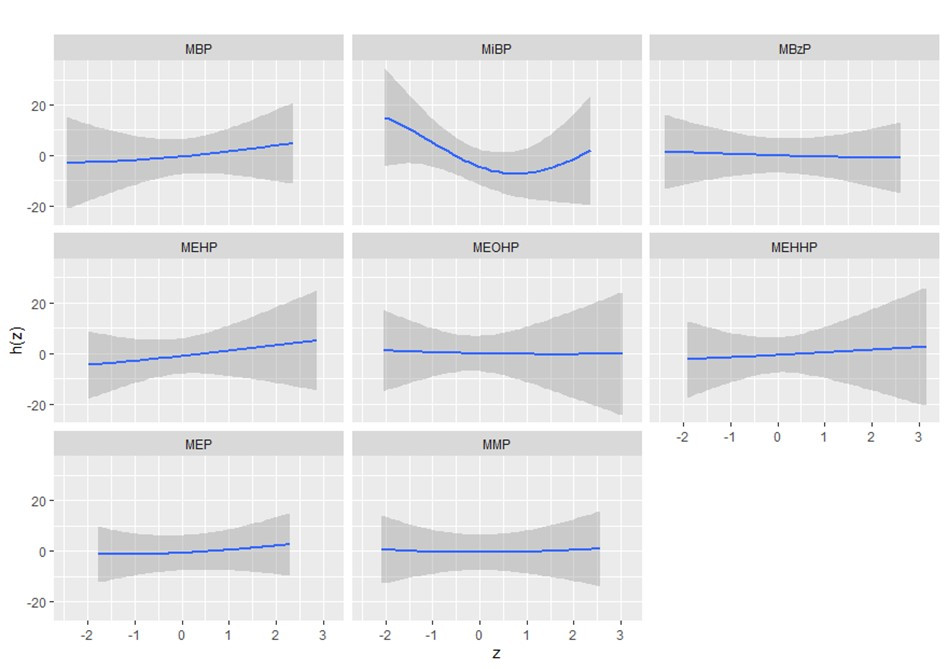


Supplementary Figure S7. Associations of a one-IQR difference between individual urinary phthalate metabolites and ultrasound PV with all other phthalates held constant at the 25^th^, 50^th^, and 75^th^ percentiles, adjusted for maternal specific gravity, maternal age, maternal BMI, maternal cigarette smoke, maternal education level, and gestational age at enrollment, among male infants born to women who identified as Black in Charleston, South Carolina (n=83)


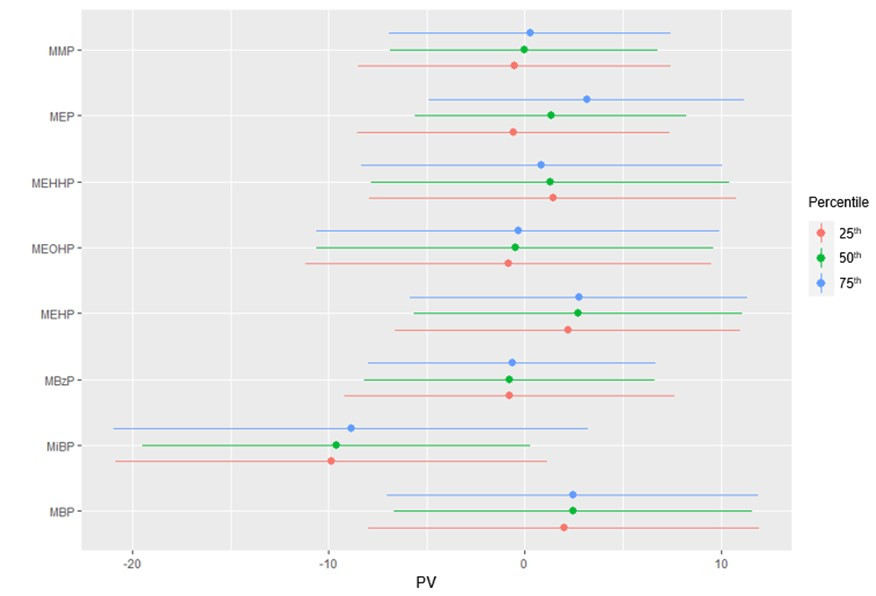


Supplementary Figure S8. Differences in ultrasound PW associated with percentiles of a urinary phthalate metabolites mixture (with the 25^th^ percentile as the reference group), adjusted for maternal specific gravity, maternal age, maternal BMI, maternal cigarette smoke, maternal education level, and gestational age at enrollment, among male infants born to women in Charleston, South Carolina, by racial identity (n=178)


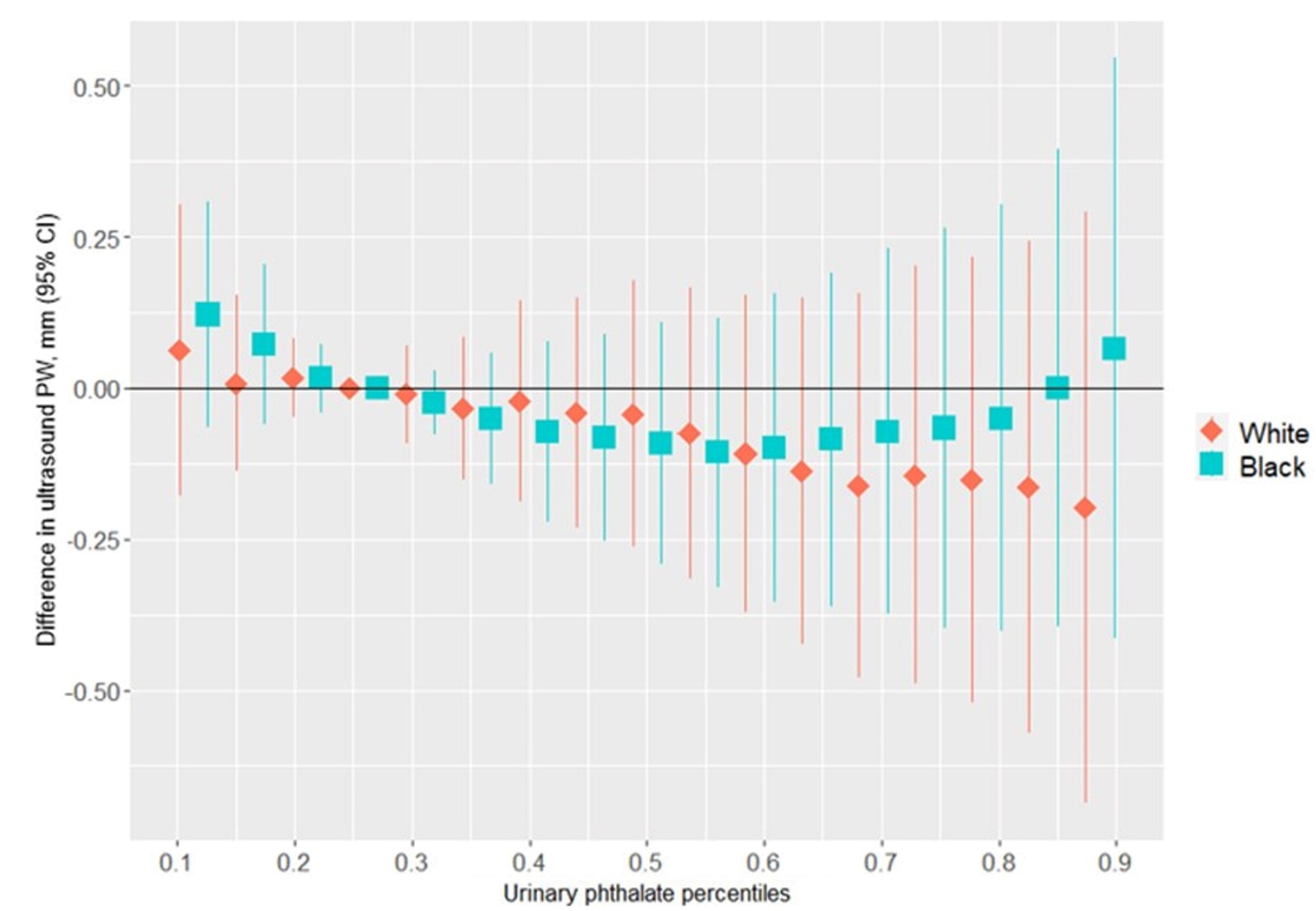


Supplementary Figure S9. Differences in ultrasound PL associated with percentiles of a urinary phthalate metabolites mixture (with the 25^th^ percentile as the reference group), adjusted for maternal specific gravity, maternal age, maternal BMI, maternal cigarette smoke, maternal education level, and gestational age at enrollment, among male infants born to women in Charleston, South Carolina, by racial identity (n=178)


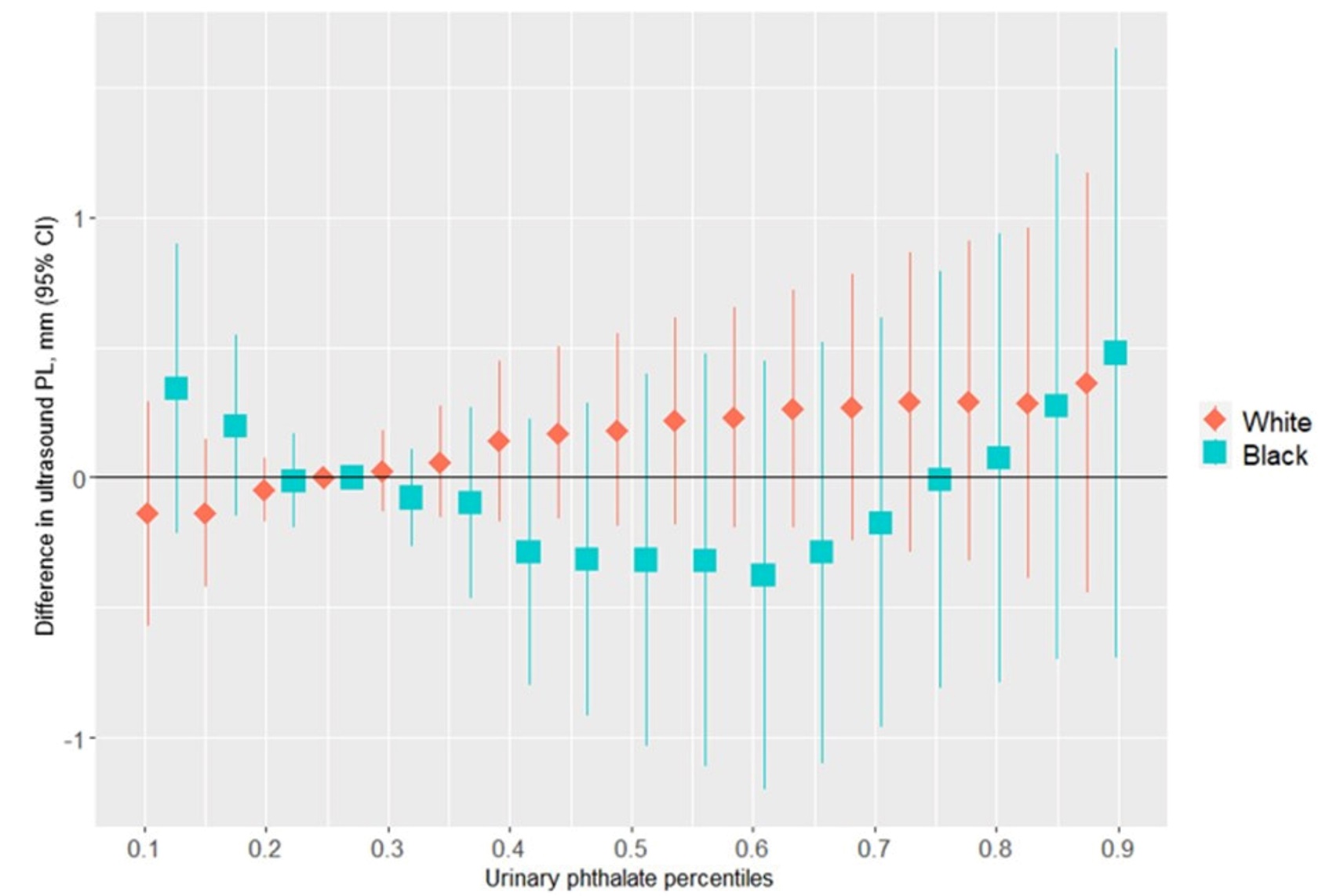


Supplementary Figure S10. Univariate exposure response plots between urinary phthalate metabolites and postnatal ACD, adjusted for maternal specific gravity, maternal age, maternal BMI, maternal cigarette smoke, maternal education level, and birth weight z-score among female infants born to women who identified as Black, in Charleston South Carolina (n=67)


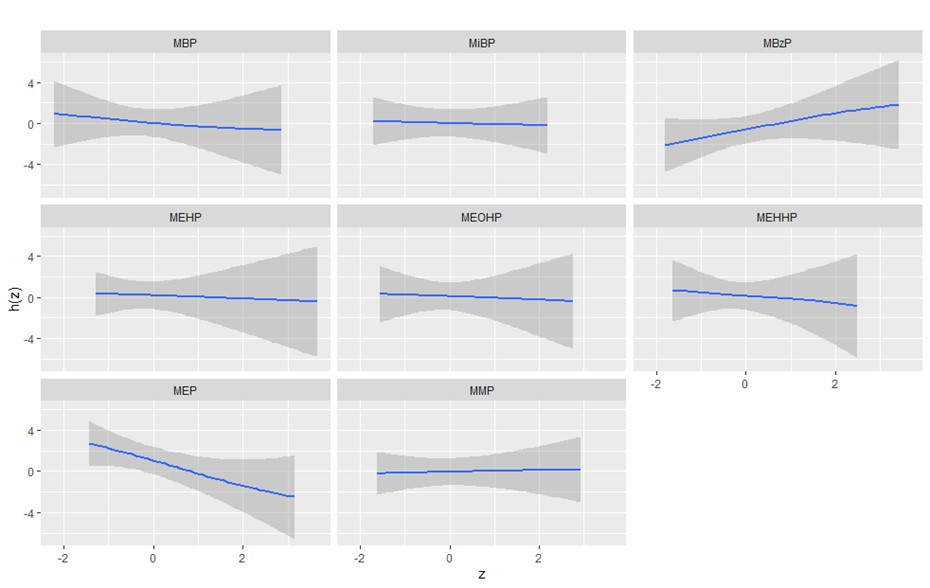


Supplementary Figure S11. Associations of a one-IQR difference between individual urinary phthalate metabolites and postnatal ACD with all other phthalates held constant at the 25^th^, 50^th^, and 75^th^ percentiles, adjusted for maternal specific gravity, maternal age, maternal BMI, maternal cigarette smoke, maternal education level, and birth weight z-score among female infants born to women who identified as Black in Charleston, South Carolina (n=67)


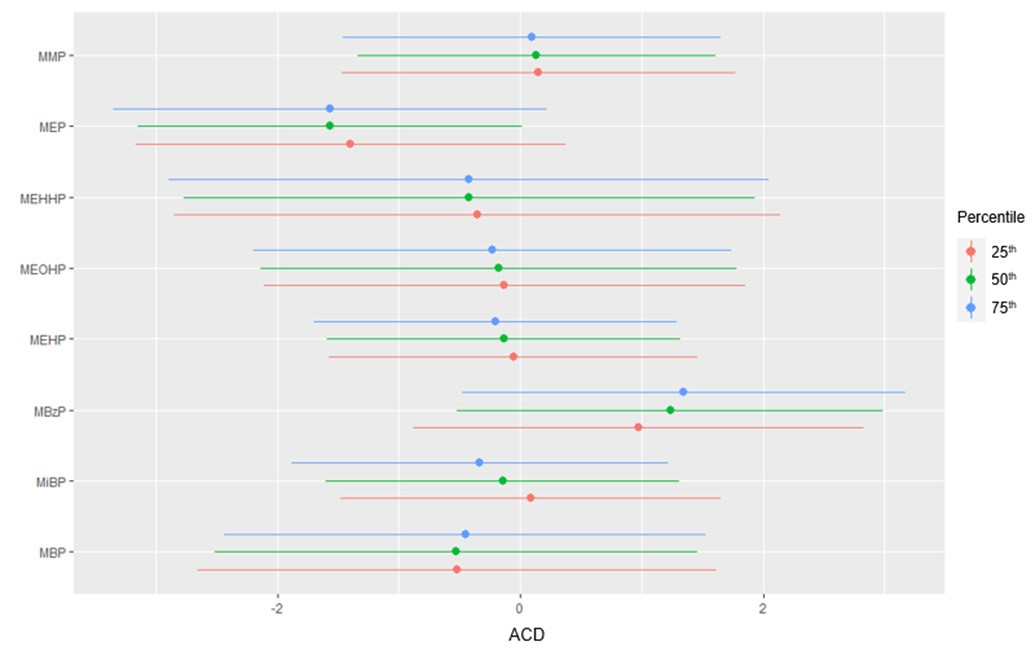


Supplementary Figure S12. Differences in postnatal AFD associated with percentiles of a urinary phthalate metabolites mixture (with the 25^th^ percentile as the reference group), adjusted for maternal specific gravity, maternal age, maternal BMI, maternal cigarette smoke, maternal education level, and gestational age at enrollment, among female infants born to women in Charleston, South Carolina, by racial identity (n=128)


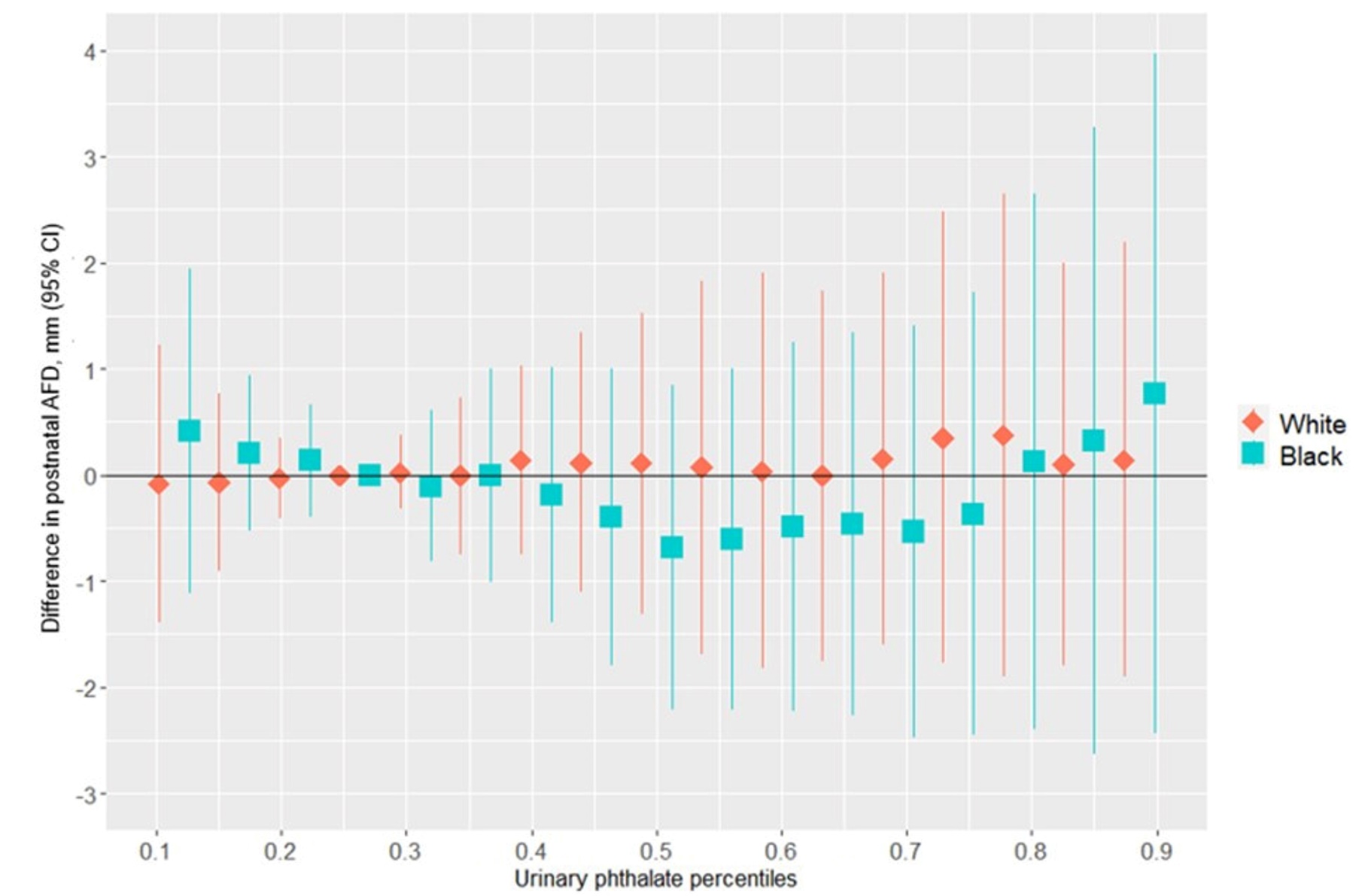

Supplement: Supplementary file 1 [file Datasheet1.docx]
